# The influence of antibody humanization on shark variable domain (VNAR) binding site ensembles

**DOI:** 10.3389/fimmu.2022.953917

**Published:** 2022-09-02

**Authors:** Monica L. Fernández-Quintero, Anna-Lena M. Fischer, Janik Kokot, Franz Waibl, Clarissa A. Seidler, Klaus R. Liedl

**Affiliations:** Department of General, Inorganic and Theoretical Chemistry, and Center for Molecular Biosciences Innsbruck (CMBI), University of Innsbruck, Innsbruck, Austria

**Keywords:** shark, VNAR, novel biotherapeutic formats, humanization, molecular dynamics simulations, hydrophobicity

## Abstract

Sharks and other cartilaginous fish produce new antigen receptor (IgNAR) antibodies, as key part of their humoral immune response and are the phylogenetically oldest living organisms that possess an immunoglobulin (Ig)-based adaptive immune system. IgNAR antibodies are naturally occurring heavy-chain-only antibodies, that recognize antigens with their single domain variable regions (VNARs). In this study, we structurally and biophysically elucidate the effect of antibody humanization of a previously published spiny dogfish VNAR (parent E06), which binds with high affinity to the human serum albumin (HSA). We analyze different humanization variants together with the parental E06 VNAR and the human Vκ1 light chain germline DPK9 antibody to characterize the influence of point mutations in the framework and the antigen binding site on the specificity of VNARs as reported by Kovalenko et al. We find substantially higher flexibility in the humanized variants, reflected in a broader conformational space and a higher conformational entropy, as well as population shifts of the dominant binding site ensembles in solution. A further variant, in which some mutations are reverted, largely restores the conformational stability and the dominant binding minimum of the parent E06. We also identify differences in surface hydrophobicity between the human Vκ1 light chain germline DPK9 antibody, the parent VNAR E06 and the humanized variants. Additional simulations of VNAR-HSA complexes of the parent E06 VNAR and a humanized variant reveal that the parent VNAR features a substantially stronger network of stabilizing interactions. Thus, we conclude that a structural and dynamic understanding of the VNAR binding site upon humanization is a key aspect in antibody humanization.

## Graphical Abstract

VNAR humanization - identifying the critical residues determining the antigen binding site and consequently influencing antigen recognition.
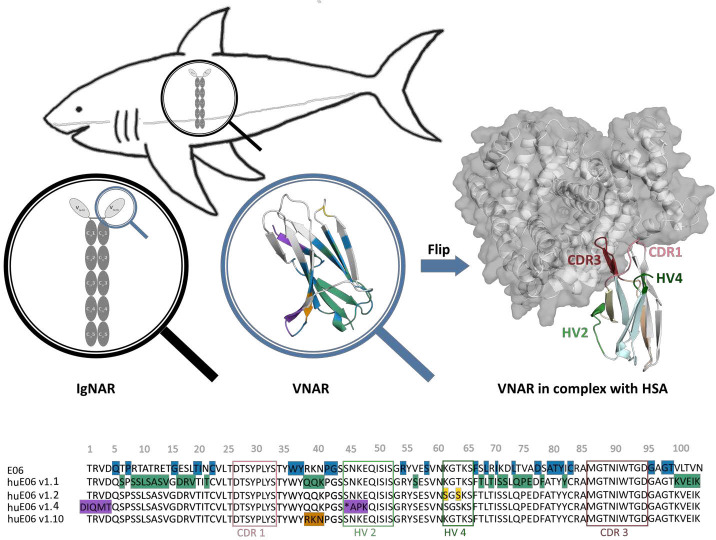

## Introduction

**Figure 1:**
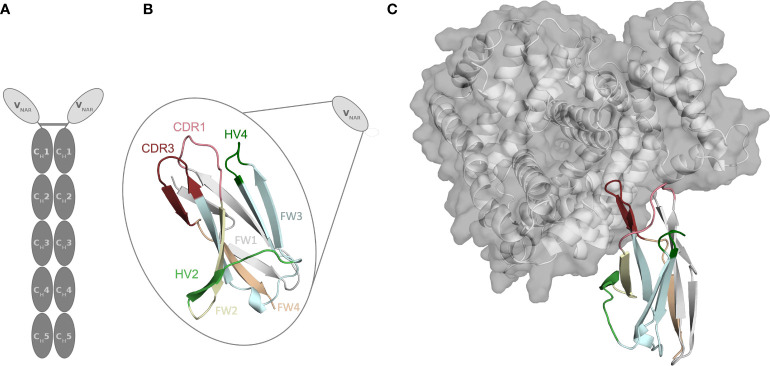
Structure of the parent E06 VNAR (PDB: 4HGK) with and without the antigen present. **(A)** Schematic representation of a new antigen receptor (IgNAR). The five constant domain dimers are shown in dark grey, while the variable single-domains (VNARs) are depicted in light grey. **(B)** Schematic and structural representation of the parent VNAR E06. The CDR1, and CDR3 loops are colored in pink and red, respectively. The FW1, FW2, FW3, and FW4 are illustrated in light grey, pale green, aquamarine, and pare orange, respectively. **(C)** Structure of the parent E06 VNAR in complex with the antigen HSA.

Approximately 500 million years ago, cartilaginous fish diverged from a common ancestor with other jawed vertebrates and can be divided into two extant subclasses, the holocephalans (chimeras and ratfish) and the elasmobranchs (sharks, rays and skates) (1–3). Cartilaginous fish are the phylogenetically oldest group of animals having an adaptive immune system, based on immunoglobulins (Ig), T-cell receptors and major histocompatibility complexes (MHC) (4–7). However, cartilaginous fish developed unique structural and immunological features and provide valuable insights in the evolution of the immune system (4, 8). Shark antibodies have evolved under challenging conditions, i.e., the high concentration of the protein denaturant urea in the blood serum and are therefore believed to be very stable (4, 9). The humoral immune response of sharks and their relatives comprises three Ig isotypes, namely IgM, IgW and so-called Ig new antigen receptor (IgNAR) (10). While the IgM and IgW are conventional heavy-light chain isotypes, the third atypical isotype IgNAR is a heavy-chain-only disulfide-bonded homodimer that does not pair with a light chain. The IgNAR consists of two identical heavy chains, which can be subdivided into two unpaired variable (VNAR) domains and five constant domain dimers (C_H_1-C_H_5) (**Figure 1**) (8, 11, 12). A schematic IgNAR structure, as well as an example of a VNAR crystal structure, is shown in **Figures 1A** **, B** (PDB accession code: 4HGK) (13). VNARs are small in size, soluble, exist as independent stable single-domains in solution and are known for their ability to bind and recognize a variety of antigens, including buried epitopes that are not accessible by conventional antibody variable domains (14–16). Due to their unique biophysical properties and characteristics, which are only shared by camelid single-chain antibodies, VNARs have received increasing attention as highly versatile proteins, which contribute to the success to date of alternative scaffolds and makes them attractive novel biotherapeutic proteins (14, 15, 17, 18). VNARs are structurally more similar to variable light chain and variable T-cell receptor domains than to variable heavy chain domains (19, 20). However, they contain only two complementarity determining region (CDR) loops, lacking the CDR2 loop and two β-strands. Instead, they contain other CDR like regions, namely the hypervariable region 2 (HV2), and the hypervariable region 4 (HV4), which reveal an elevated rate of somatic hypermutations. To compensate for the reduced size, the binding site features a long and extended CDR3 loop, which has the highest diversity in length, sequence, and structure (18, 21).

**Figure 2:**
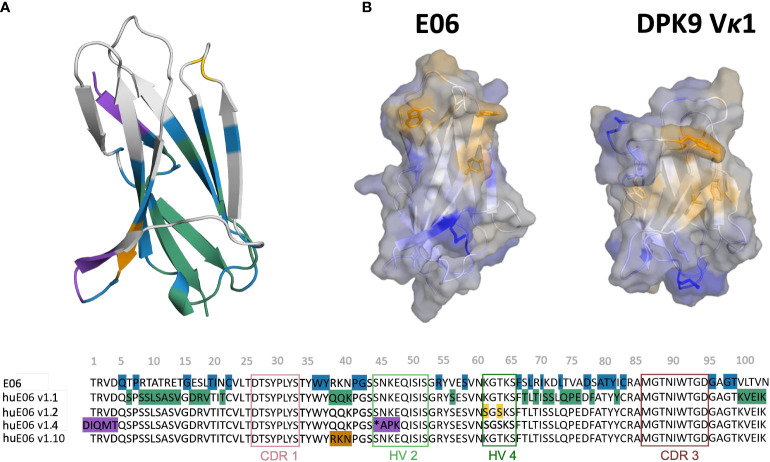
Sequence alignment highlighting the differences between the VNAR variants. **(A)** Color-coded sequence alignment showing the introduced changes upon antibody humanization. The changes in the sequences are also highlighted in the structure. The residues that are identical with the DPK9 germline are colored in blue, the mutated residues in variant huE06 v1.1 are colored in green. Additional mutations of variants huE06 v1.2, v1.4 are depicted in yellow and purple, respectively. The huE06 v1.10 variant, restoring critical ‘RKN’ motif, is shown in orange. **(B)** Surface hydrophobicity of the parent VNAR E06 and the human DPK9 light chain variable domain, as assigned by the Wimley and White the hydrophobicity scale (26).

Another critical determinant of the structural diversity of VNARs is that they have further disulfide bonds in the CDRs and the framework region in addition to the canonical cysteine residues (Cys23-Cys88 – Kabat nomenclature) in the framework. Based on the number and position of additional cysteine residues, four types of naturally occurring IgNAR variable domains have been classified (4, 9, 12, 22, 23). Type I VNARs, which can be found in nurse sharks, contain two cysteines in CDR3 and two more in the framework regions 2 and 4. In contrast, type II and III VNARs have an additional cysteine pair to link CDR1 and CDR3 loops. Type III VNARs are predominantly present in neonatal shark development but have subsequently been found in adult spiny dogfish sharks and bamboo sharks. Structurally, these antibodies are characterized by a conserved tryptophan residue in the CDR1 loop and limited CDR3 loop diversity, however, the functional role of these antibodies remains still elusive. Type IV VNARs, which are primarily found in dogfish sharks, wobbegong, small-spotted catsharks and bamboo sharks contain only the two canonical cysteine residues (9, 13, 24, 25). In this study, we investigate the consequences and effects of antibody humanization of the spiny dogfish shark VNAR E06 (**Figure 2**). We thermodynamically and kinetically characterize the conformational diversity and the respective ensembles in solution of the E06 VNAR and compare them with different humanization variants published by Kovalenko et al., and the germline light chain Vκ1 antibody, DPK9 (13). The investigated variants have been humanized by converting over 60% of non-CDR residues to those of the human germline and the resulting antibodies have mostly retained the specificity and affinity of the parent E06. For the first humanization variant (huE06 v1.1), the E06 VNAR has been humanized by replacing 63.5% of the framework residues (FW) of the VNAR E06, i.e., FW1 (residues 6-21), FW2 (residues 38-40), FW3 (residues 66-82) and FW4 (residues 99-103), with residues from the human DPK9, while keeping the original shark residues for the first four N-terminal residues, CDR1, CDR3, HV2 and HV4 loop residues (**Figure 2A**). The DPK9 (Vκ1) light chain was chosen for humanization, as it is one of the most stable and well-expressed human frameworks, that shares significant structural homology with E06 (27). The huE06 v1.2 variant was generated by introducing additional mutations in the HV4 loop. Additionally, mutations in the HV2 loop and in the N-terminus towards DPK9 were made to obtain huE06 v1.4. The variant E06 v1.10 was obtained by restoring critical contact residues (namely residues 38-40) with the antigen in FW2 (**Figure 2A**), which resulted in improved binding properties compared to E06 v1.1. Additionally, two crystal structures of the parental E06 VNAR and the huE06 v1.1 variant in complex with the HSA antigen were available (13). The two complex crystal structures show that not only CDR loops are responsible for interacting with the antigen, but that also several framework residues are in contact to HSA.

## Results

Where possible, we started our simulations from crystal structures. For, the other variants including the DPK9 (Vκ1) light chain variable domain, we generated models by using “AlphaFold2” (**Table 1**) (13, 28). In **Figure 2B**, we present the surface hydrophobicity of the parent VNAR E06 and the human DPK9 light chain variable domain, as assigned using the hydrophobicity scale by Wimley and White (26). It shows that the DPK9 variable domain displays a hydrophobic patch at the center of the interface, which corresponds to the contact area with the respective paired heavy chain. This hydrophobic patch in the DPK9 interface is replaced by a more hydrophilic surface in the E06 VNAR.

**Table 1:** Overview of the investigated variants with the aggregated simulation times.

Variants	Starting structures	Simulation time/µs
**E06**	PDB: 4HGK	11.9
**huE06 v1.1**	PDB: 4HGM	28.2
**huE06 v1.2**	AlphaFold2	17.4
**huE06 v1.4**	AlphaFold2	43.1
**huE06 v1.10**	AlphaFold2	20.0
**DPK9 (Vκ1)**	AlphaFold2	33.8

To characterize the conformational diversity of the E06 VNAR and the humanized variants in solution, we applied a protocol using the enhanced sampling technique metadynamics in combination with classical molecular dynamics (MD) simulations, to overcome the limitations of conformational sampling imposed by high energy barriers. The aggregated simulation time for each variant is summarized in **Table 1**.

**Figure 3:**
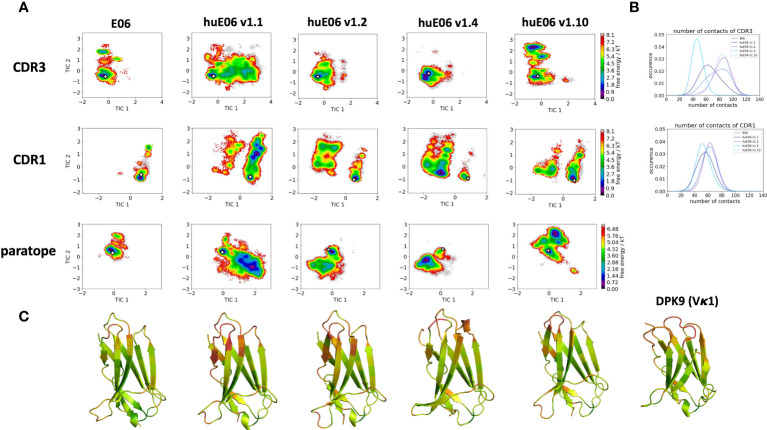
Free energy landscapes of the CDR1, CDR3 and the paratope (CDR1, CDR3 and HV2 loops), intradomain contact distributions of the individual CDR loops and dihedral entropies projected onto the VNAR structures as measure for flexibility. **(A)** Free energy surfaces of the CDR1, CDR3 and the paratope for the parent E06 VNAR and the different humanization variants in the same coordinate system, respectively. The available crystal structures are depicted as white diamonds, while the models are illustrated as white circles. **(B)** Contacts per frame distributions of the CDR1 and CDR3 loops. **(C)** Residue-wise dihedral entropies mapped onto the respective structures (red – high flexibility, green – low flexibility).

**Figure 4:**
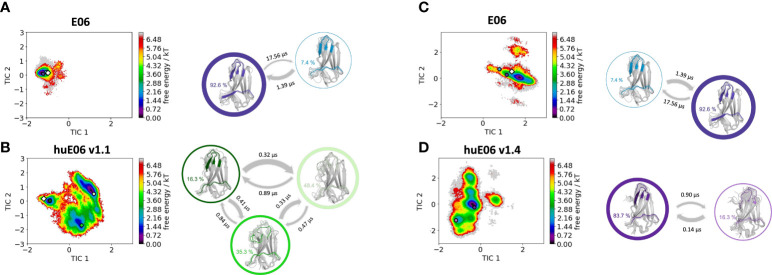
Kinetic and thermodynamic VNAR binding site ensemble characterization of the parent E06 VNAR and the humanized variants huE06 v1.1 and huE06 v1.4. **(A, B)** Free energy landscapes with the respective state probabilities, transition timescales and macrostate representatives of the parent E06 and the humanized variant huE06 v1.1 in the same coordinate system. The crystal structures are depicted as white diamonds. Macrostate representatives are projected into the free energy landscape as dots, color-coded according to the structures on the right. The thickness of the arrows denotes the transition timescale and the width of the surrounding circle represents the state population. **(C, D)** Free energy surfaces with the respective state probabilities, transition timescales and macrostate representatives of the parent E06 and the humanized variant huE06 v1.4 in the same coordinate system. The available crystal structure is depicted as white diamond.

Moreover, we wanted to characterize and quantify the conformational diversity in the different humanized variants. **Figure 3A** shows the time-lagged independent component analysis (tICA) plots of the CDR3, CDR1 and of the whole paratope (comprising CDR1, CDR3 and the HV2 loop). tICA is a dimensionality-reduction technique, which detects the slowest-relaxing degrees of freedom. The free energy landscapes of the paratope clearly show that the parent E06 VNAR is less flexible than the humanized variants. This difference in conformational diversity is also reflected in the free energy surfaces of the individual CDR1 and CDR3 loops, which reveal different conformational states in solution. **Figure 3B** shows histograms of the number of intradomain contacts of the CDR3 and the CDR1, respectively. There is a clear reduction of the number of contacts in those variants which also show higher flexibility in the free energy surfaces. In agreement with experimental affinity and specificity measures determined by Kovalenko et al. (13), in which huE06 v1.10 is the closest to the parent E06 VNAR, we find that the parent E06 and the huE06 v1.10 display the highest number of intradomain contacts per frame. **Figure 3C** visualizes the residue-wise dihedral entropies projected onto the respective E06 VNAR and the humanized variant structures. We find clear differences in the dihedral entropy of the CDR loops and the HV2 and HV4 loops between the different variants and the parent VNAR. The huE06 v1.10 variant and the parent VNAR show the most limited flexibility, while the variant huE06 v1.4 reveals together with the germline DPK9 variable domain and the huE06 v1.1 variant the highest conformational diversity. A direct comparison of the CDR3 loop conformational spaces of the parent, the humanized variants, and the germline DPK9 variable domain is shown in SI **Figure S1**. The germline CDR3 loop reveals a high flexibility, reflected in a broader conformational space and a different dominant minimum in solution. To reconstruct thermodynamics and kinetics of different loop rearrangements, we built Markov-state models of the paratope based on the backbone torsions of the CDR1, CDR3, and HV2 (**Figure 4**). **Figure 4** compares the parent E06 with the variant huE06 v1.1 (**Figures 4A** **, B**) and with the variant huE06 v1.4 (**Figures 4C** **, D**) in the same coordinate system, respectively. We find overlaps of the conformational spaces of the parent and the humanized variants; however, the variants and the parent differ in flexibility and in their state probabilities. While the available complex X-ray structure (depicted as white diamond) lies in the dominant minimum in solution (92% probability) of the parent E06 VNAR, the probability of this state is substantially reduced to 16% in the huE06 v1.1 variant. The difference is more drastic for the comparison of the parent E06 VNAR with the huE06 v1.4.

**Figure 5:**
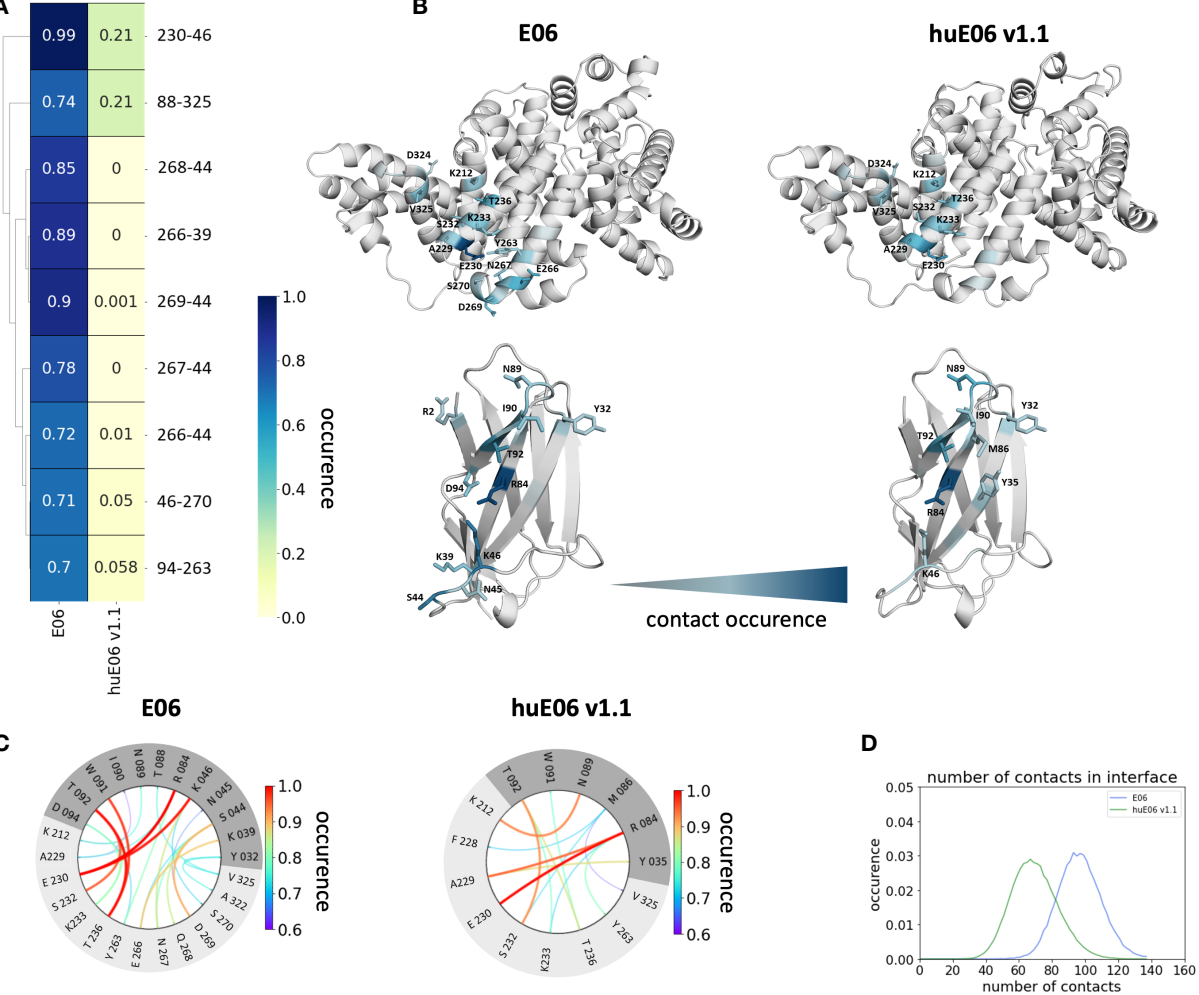
Contact analysis of the HSA antigen with the parent E06 VNAR and the huE06 v1.1. **(A)** Interaction fingerprint visualization of the contacts between antibody and antigen, depicts the differences in the contact frequencies between the parent and the humanized variant. **(B)** Interacting residues between antibody and antigen color-coded according to their occurrence. **(C)** Interaction flareplots between antibody (dark grey) and antigen HSA (light grey). The color-coding of the lines in the flareplots corresponds to the occurrence of the contacts. **(D)** Contacts per frame distributions for the interactions of the parent and the humanized variant with the antigen. The distribution of the parent is depicted in blue, the distribution of huE06 v1.1 is illustrated in green.

In line with the results obtained for huE06 v1.1, we observe a bigger conformational landscape and find a substantially reduced probability for the binding competent state of the huE06 v1.4 (16%). As described in the methods section, we also performed simulations in complex with the antigen, as both available crystal structures were in complex with the HSA antigen. We do not only find a substantial increase of flexibility in the antigen-binding site for the huE06 v1.1, compared to the parent E06 VNAR, but we also find substantial differences in the interaction networks. **Figure 5** shows the interaction patterns of the parent E06-HSA complex compared with the huE06 v1.1-HSA complex. We observe a shift in the contact per frame distributions towards a lower number of contacts for the huE06 v1.1 variant (**Figure 5D**). This substantial reduction in the interaction network is also visualized in the antibody-antigen interaction fingerprints and flareplots (**Figures 5A, C**). The main differences in antibody-antigen interactions are also visualized in the interaction fingerprint plots, which provide a clustering of interactions based on their contact frequencies. These findings agree with the reduced binding affinity and specificity of the huE06 v1.1 to the HSA compared to the parent E06 VNAR and indicate that they result from missing interactions with the HV2 loop and the framework.

## Discussion

The rise of antibodies as biotherapeutic proteins has motivated numerous studies to characterize and understand the antibody binding interface as a pre-requisite for rational antibody design and engineering (29–35). Compared to conventional antibodies, small antibodies, such as nanobodies and VNARs, offer various advantages and reveal features that are desirable for drug discovery, i.e., higher stability and solubility. Additionally, they can work inside cells, recognize cryptic and buried epitopes and due to their small size wend into tissues (21). Furthermore, it has also been reported that VNARs can be used as blood-brain barrier (BBB) shuttles or transporter molecules (36).

Thus, to design and engineer these outstanding proteins, it is crucial to characterize the peculiar antibody binding site of VNARs structurally and dynamically and to elucidate the antibody-antigen recognition mechanism. In this study, we thermodynamically and kinetically characterize the consequences of humanization on VNAR binding site ensembles in solution and provide a description of the fundamental factors that contribute to antigen binding. Engineering efforts focus on reducing undesirable immunogenic responses by antibody humanization, thereby increasing the identity of non-human antibodies and scaffolds to common human antibody sequences (37–41). The main challenge in humanizing antibodies is to maintain the full biological function, which is reflected in a high binding affinity and specificity to reduce the risk of adverse side-effects (42). Therefore, to understand the role of the framework on the antigen binding site, it is crucial to biophysically characterize antibody paratope ensembles in solution in different stages of antibody humanization (38). Conformational rearrangements in the paratope, as well as antibody-antigen binding can occur in the nano-to-millisecond timescale, which exceeds routinely performed simulation times by far (43, 44). To enhance the sampling efficiency, we use metadynamics simulations to cover the relevant conformational transitions and paratope rearrangements. **Figure 2A** shows the sequence alignment of the parent E06 VNAR with the investigated humanized variants and highlights the importance of specific framework residues for antigen recognition. **Figure 1C** shows the atypical binding mode of the antigen HSA to the VNAR, as not only the CDR loops are involved in binding the antigen, but also the HV2 and extensive framework residues.

It has already been shown that framework residues can determine the binding site ensembles and consequently influence antibody-antigen binding (13, 37, 39, 45, 46). Additionally, **Figure 2B** depicts the surface hydrophobicity of the parent E06 VNAR compared to the human germline DPK9. We find that the parent E06 VNAR reveals a more hydrophilic interface, which is believed to be responsible for the enhanced stability and the favorable biophysical characteristics (17, 18). **Figure 3** provides an overview of the CDR loop and paratope conformational spaces and clearly shows that the shape of the paratope is not solely determined by the CDR3 loop, but is actually also influenced by the CDR1 and the HV2 loops. Compared to the parent E06 VNAR, we find that in line with the decrease in specificity reported by Kovalenko et al. (13), we observe an increase in flexibility for all investigated humanized variants and the human germline DPK9 variable domain. This increase in flexibility is reflected in the broader conformational landscapes and the increased residue-wise dihedral entropies. The residue-wise dihedral entropies provide an alignment-independent measure for flexibility and thereby facilitate the comparison of flexibility hotspots within the different variants (47). The changes in flexibility underline the challenges in rationally designing antibodies, by revealing the presence of conformational substates, which are likely to have different binding properties and may result in a high entropic cost upon binding (48). These observations presented in **Figure 3** also agree with previous studies showing that affinity maturation of single-domain variable domains results in a decrease of flexibility (49–52). Apart from differences in flexibility, we observe substantial shifts of the contact per frame distributions (**Figure 3B**), in particular for the humanized variants huE06 v1.1, v1.2 and v1.4. Thus, we find that not only the direct interactions with the antigen are influenced by humanization (**Figure 5**), but also the intramolecular interaction network. Therefore, the higher flexibility of the variants can also be explained by a weaker intramolecular interaction network. The lowest number of contacts per frame can be found for the huE06 v1.4 variant, which lacks a charged critical hydrogen bond interaction of residue K64 in the HV4 loop and residue Y29 in the CDR1 loop and forms instead a less probable interaction between residues T27-Y29 in the CDR1 loop. The huE06 v1.10 variant on the other hand restores the ‘RKN’ motif located in FW2, which is crucial for antigen-binding and reveals therefore a highly similar intramolecular interaction network compared to the parent E06 VNAR. The Markov-state models in **Figure 4** compare the parent E06 VNAR paratope states with the humanized variants huE06 v1.1 and huE06 v1.4. We find that the binding competent conformation is present in all investigated variants, however with reduced probability. These findings support the idea that the decrease in specificity is accompanied by a population shift, reflected in different dominant states in solution as a consequence of antibody humanization. The simulations with the antigen HSA reveal that the huE06 v1.1 displays also in complex an increase in flexibility compared to the parent E06 VNAR-HSA complex (SI **Figure S2**). **Figure 5** shows the interaction profiles and networks of the parent E06 VNAR and the huE06 v1.1 variant and reveals a substantially reduced number of interactions for the huE06 v1.1. The higher flexibility of the huE06 v1.1 variant can be explained by the absence of the stabilizing salt bridge interaction between residue K46 in the HV2 loop with residue E230 in the antigen and the hydrogen bond interaction of residue W91 in the CDR3 loop with residue T236.

## Conclusion

In this study, we structurally and functionally characterized the antigen-binding site of five VNARs upon antibody humanization. We observed, in line with the findings of Kovalenko et al., that not solely the CDR1 and CDR3 loops are critical for determining the shape of the antigen-binding site, but that also the HV2 loop and the FW2 are critical for antigen recognition. The germline DPK9 as well as the humanized variants reveal a higher flexibility, which is reflected in a higher conformational entropy, a broader conformational space and substantial population shifts of the dominant binding site ensembles in solution. The huE06 v1.10 variant restores three critical framework residues and retains the conformational stability and the dominant binding minimum of the parent. Additionally, the simulations in complex with the antigen reveal that the huE06 v1.1 variant shows a higher flexibility and variability in the antigen-binding site compared to the parent E06 VNAR. Apart from the higher conformational variability, the VNAR-HSA complexes differ in the duration and number of antibody-antigen interactions. The increase in flexibility upon antibody humanization can be explained by a lack of stabilizing interactions, due to changes in the framework. Thus, we conclude that the dynamic and structural characterization of VNAR binding site ensembles allows to identify the key determinants for antigen recognition and can guide antibody humanization efforts.

## Methods

A previously published method characterizing the CDR loop ensembles in solution (32, 33, 38, 50, 51, 53, 54) was used to investigate the conformational diversity of the paratope loops of VNAR humanization variants both with and without the antigen bound (13). Experimental structure information was available for the parent E06 VNAR and the humanized variant huE06 v1.1, which were crystallized with the antigen HSA. The PDB accession codes for the parent E06 and the huE06 v1.1 are 4HGK and 4HGM, respectively. For the other investigated variants and the human Vκ1 germline DPK9, we used AlphaFold2 to predict the structures (28). The available X-ray structures and the models were used as starting structures for molecular dynamics simulations. The starting structures for simulations were prepared in MOE (Molecular Operating Environment, Chemical Computing Group, version 2020.09) using the Protonate3D tool (55, 56). To neutralize the charges we used the uniform background charge (57, 58). Using the tleap tool of the AmberTools20 (57, 59) package, the structures were soaked in cubic water boxes of TIP3P water molecules with a minimum wall distance of 10 Å to the protein (60–62). For all simulations, parameters of the AMBER force field 14SB were used (63). The VNAR variants were carefully equilibrated using a multistep equilibration protocol (64).

### Metadynamics simulations

To enhance the sampling of the conformational space, well-tempered metadynamics simulations (65–68) were performed in GROMACS (69, 70) with the PLUMED 2 implementation (71). As collective variables, we used a linear combination of sine and cosine of the ψ torsion angles of the CDR1 and CDR3 loops calculated with functions MATHEVAL and COMBINE implemented in PLUMED 2 (71). As discussed previously the ψ torsion angle captures conformational transitions comprehensively (72). The decision to include the ψ torsion angles of these two loops is based on their strong involvement in the binding to the antigen as evident from the X-ray structure of the complex. The simulations were performed at 300 K in an NpT ensemble using the velocity rescaling algorithm and a Parrinello-Rahman barostat (73, 74). For the metadynamics simulations, we used a Gaussian height of 10.0 kJ/mol and a width of 0.3 radian. Gaussian deposition occurred every 1000 steps and a biasfactor of 10 was used. 1 µs metadynamics simulations were performed for the parent E06 VNAR, the humanized variants and the DPK9 human variable domain without the antigen. As the available X-ray structures for the parent E06 and the huE06 v1.1 were crystallized with the antigen present, we also performed 1 µs of metadynamics simulations in complex with the antigen for these two systems. The resulting trajectories were clustered in cpptraj (57, 59) by using the average linkage hierarchical clustering algorithm with a distance cut-off criterion of 1.3 Å resulting in a large number of clusters (**Table 1**). The cluster representatives for the parent and the humanized variants both with and without the antigen present were equilibrated and simulated for 100 ns each using the AMBER 20 simulation package (57).

### Molecular dynamics simulations

Molecular dynamics simulations were performed in an NpT ensemble using pmemd.cuda (75). Bonds involving hydrogen atoms were restrained by applying the SHAKE algorithm (76), allowing a time step of 2 fs. Atmospheric pressure of the system was preserved by weak coupling to an external bath using the Berendsen algorithm (77). The Langevin thermostat (78, 79) was used to maintain the temperature during simulations at 300 K.

Based on the backbone torsion of the CDR1, CDR3 and HV2 loops, a time-lagged independent component analysis (tICA) was performed using the python library PyEMMA 2 employing a lag time of 10 ns (80, 81). Thermodynamics and kinetics were calculated from a Markov-state model (82, 83) by using PyEMMA 2, which uses the k-means clustering algorithm (84) to define microstates and the PCCA+ clustering algorithm (85) to coarse-grain the microstates into macrostates. PCCA+ is a spectral clustering method, which discretizes the sampled conformational space based on the eigenvectors of the transition matrix. The sampling efficiency and the reliability of the Markov-state model (e.g., defining optimal feature mappings) has been evaluated with the Chapman-Kolmogorov test (86, 87), by using the variational approach for Markov processes (88) and by taking into account the fraction of states used, as the network states must be fully connected to calculate probabilities of transitions and the relative equilibrium probabilities. To capture and quantify the kinetically relevant loop rearrangements of the VNAR variants we constructed Markov-state models based on the backbone torsions of the CDR1, CDR3 and HV2 loops, defined 100 microstates using the k-means clustering algorithm and applied a lag time of 15 ns. The images presented in this paper were created by using the PyMOL molecular graphics system (89).

### Dihedral entropies

We calculated the residue-wise dihedral entropies with the recently published X-entropy python package, which calculates the entropy of a given dihedral angle distribution (47). This approach uses a Gaussian kernel density estimation (KDE) with a plug-in bandwidth selection, which is fully implemented in C++ and parallelized with OpenMP. The obtained residue-wise dihedral entropies were projected onto the respective structures.

### Contacts

The GetContacts (90) tool was used to quantify the interactions occurring in the simulations. To generate the contact per frame histograms, we chose a bin width of 2. Inspired by their visualization tools a new script was developed. The script includes “interaction fingerprints” which facilitate the comparison of interactions among multiple systems as well as “flareplots” to visualize the interaction networks of individual systems. The “interaction fingerprint” plots are based on a hierarchical clustering on the data to compare the contact frequencies of different systems. We used as clustering criterion the contact frequencies and applied the cut-off 0.5. In the fingerprint plots interacting residues are connected *via* a line that is colored according to the frequency of the interaction. The script as well as a short introduction is provided on our GitHub (https://github.com/liedllab/GetContacts_analysis).

## Data availability statement

The original contributions presented in the study are included in the article/**Supplementary Material**. Further inquiries can be directed to the corresponding author

## Author contributions

MF-Q performed research, wrote the manuscript. A-LF performed research and analyzed data. JK performed research. FW analyzed data and contributed in writing the manuscript. CS analyzed data. KL supervised the research. All authors contributed to writing the manuscript.

## Funding

This work was supported by the Austrian Science Fund (FWF) *via* the grants P30565, P30737 and P30402, P34518 as well as DOC 30.

## Acknowledgments

The computational results presented her have been achieved (in part) using the Vienna Scientific Cluster (VSC). We acknowledge PRACE for awarding us access to Piz Daint at CSCS, Switzerland.

## Conflict of interest

The authors declare that the research was conducted in the absence of any commercial or financial relationships that could be construed as a potential conflict of interest.

## Publisher’s note

All claims expressed in this article are solely those of the authors and do not necessarily represent those of their affiliated organizations, or those of the publisher, the editors and the reviewers. Any product that may be evaluated in this article, or claim that may be made by its manufacturer, is not guaranteed or endorsed by the publisher.
