# Supplementary material for: The influence of antibody humanization on shark variable domain (VNAR) binding site ensembles
Source: Front Immunol. 2022 Sep 2;13:953917. doi: 10.3389/fimmu.2022.953917 (PMC9514858; doi:10.3389/fimmu.2022.953917)
Supplement: Supplementary file 1 [file DataSheet_1.docx]

# Supporting information

#
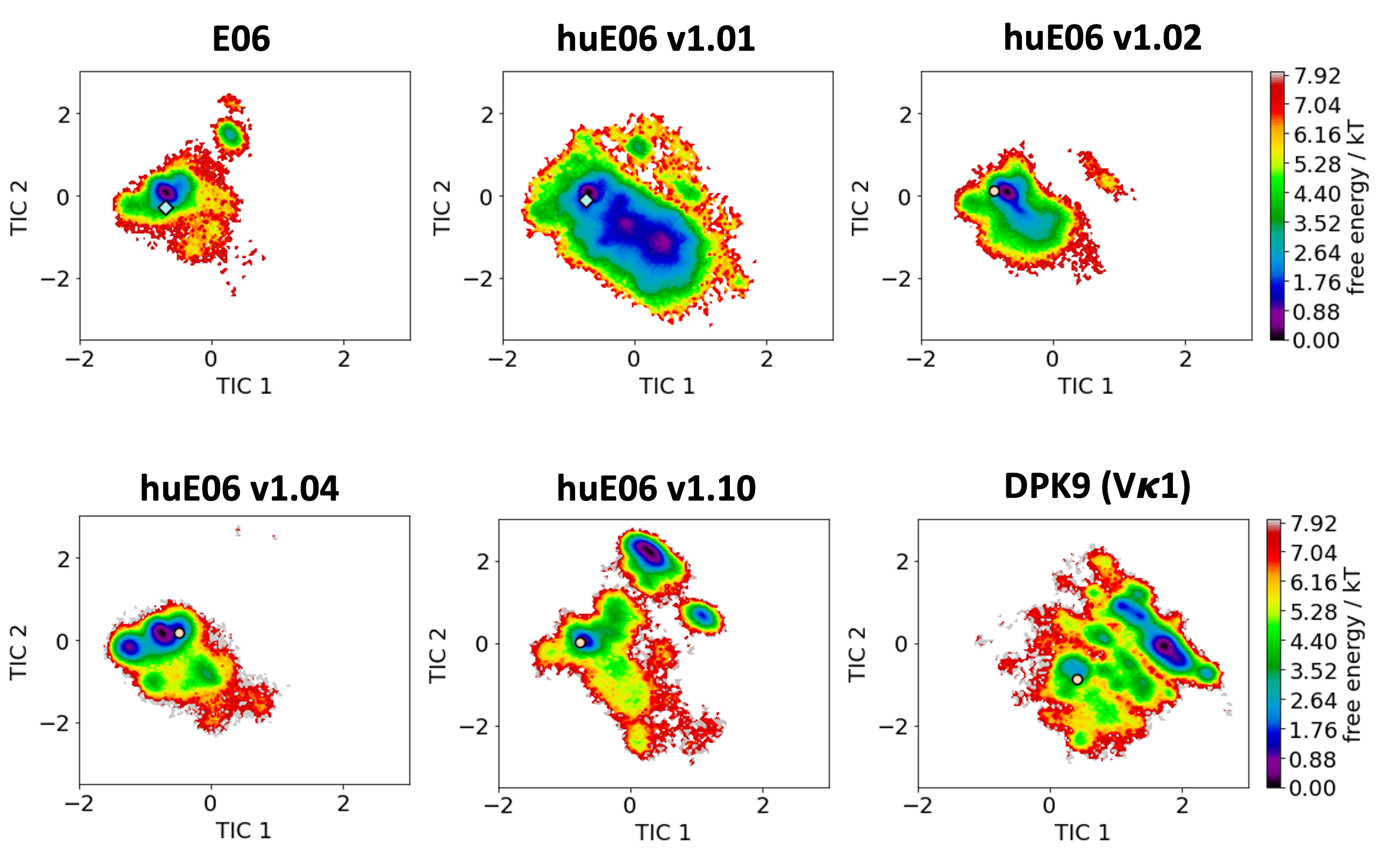


SI Figure S1: **Free energy landscapes of the CDR3 loop for the parent E06 VNAR, the humanized variants and the light chain germline DPK9 in the same coordinate system.** The available crystal structures are depicted as diamonds. For the other variants and the DPK9 germline the starting models are illustrated as yellow dots.


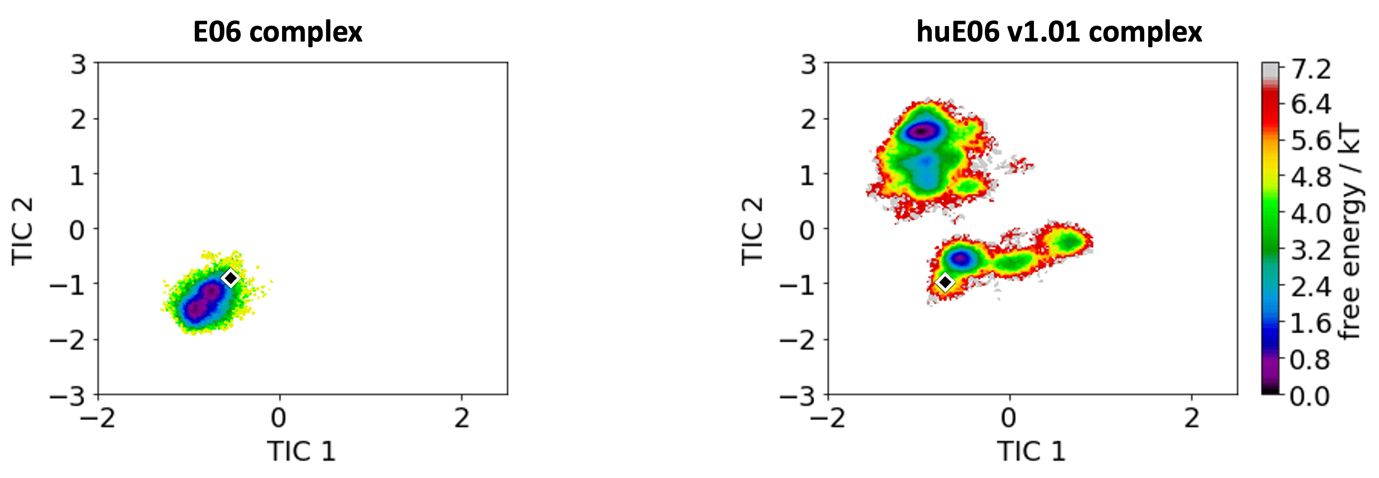


SI Figure S2: **Free energy landscapes of the paratope for the parent E06 VNAR and the huE06 v1.01 variant simulated in complex with the HSA antigen.** The available crystal structures are depicted as diamonds.
